# Supplementary material for: Ultrastructural sublaminar-specific diversity of excitatory synaptic boutons in layer 1 of the adult human temporal lobe neocortex
Source: eLife. 2025 Jul 21;13:RP99473. doi: 10.7554/eLife.99473 (PMC12279374; doi:10.7554/eLife.99473)
Supplement: Supplementary file 1. [file elife-99473-supp1.docx]

**Supplemental file 1: Comparison of the synaptic density between L1a, L1b and L4 – L6 of the human TLN.**

| **Cortical layers** | **L1a**  **mean ± SD** | **L1b**  **mean ± SD** | **Total L1**  **mean ± SD** | **L4**  **mean ± SD** | **L5**  **mean ± SD** | **L6**  **mean ± SD** |
| --- | --- | --- | --- | --- | --- | --- |
| Density of synaptic contacts/mm^3^ | 5.52*10^8^  ±  1.25*10^8^ | 5.01*10^8^  ±  1.40*10^7^ | 5.26*10^8^  ±  8.44*10^7^ | 2.37*10^6^  ±  2.19*10^6^ | 3.89 *10^8^  ±  9.12*10^8^ | 4.98 *10^7^  ±  1.85*10^7^ |

Values for L4–L6 are taken from: L4: Yakoubi et al. 2019b; L5: Yakoubi R., PhD Thesis 2023; L6: Schmuhl-Giesen et al. 2022.
