# Supplementary material for: Ultrastructural sublaminar-specific diversity of excitatory synaptic boutons in layer 1 of the adult human temporal lobe neocortex
Source: eLife. 2025 Jul 21;13:RP99473. doi: 10.7554/eLife.99473 (PMC12279374; doi:10.7554/eLife.99473)
Supplement: Supplementary file 2. [file elife-99473-supp2.docx]

**Supplemental File 2: Patient’s identity and medical background.**

| **Patient’s identity**  **and medical background** | **Gender** | **Age**  **(years)** | **Age at epilepsy onset (years)** | **Histo-**  **Pathology** | **Antiepileptic drugs (pre-op)** | **Number of**  **SBs recon-**  **structed** |
| --- | --- | --- | --- | --- | --- | --- |
| Hu_01 | **♂** | 52 | 20 | AHS | LEV, OXC, Perampanel | L1a: 63; L1b: 70 |
| Hu_02 | **♀** | 27 | 16 | Micro- and Astrogliosis | LEV, Topiramat, Zebinix | L1a: 72; L1b: 45 |
| Hu_03 | **♀** | 65 | 1 | Hippocampal sclerosis | LEV, LTG,  Primidone | L1a: 55 |
| Hu_04 | **♀** | 24 | N/A | TLE | N/A | L1b: 56 |
| Hu_05* | **♀** | 36 | 4 | GGL | LEV, LTG |  |
| Hu_06* | **♀** | 25 | 12 | AHS | LTG |  |
| Hu_07* | **♀** | 25 | 23 | GGL | Zebinix, LEV |  |
| Hu_08* | **♂** | 33 | 5 | Gliosis | LEV, CBZ |  |
| Hu_09* | **♂** | 63 | 24 | AHS | LEV, LTG, CBZ |  |

Abbreviation: AHS: Ammon's horn sclerosis; CBZ: Carbamazepine; GGL: Ganglioglioma; LEV: Levetiracetam; LTG: Lamotrigine; OXC: Oxcarbazepin; TLE: Temporal Lope Epilepsy. *Hu_05 – Hu_09 were only used to analyze the astrocytic coverage of AZs in L4.
